# Forward and Backward Walking: Multifactorial Characterization of Gait Parameters

**DOI:** 10.3390/s23104671

**Published:** 2023-05-11

**Authors:** Lucia Donno, Cecilia Monoli, Carlo Albino Frigo, Manuela Galli

**Affiliations:** 1 Department of Electronics, Information and Bioengineering, Politecnico di Milano, Piazza Leonardo da Vinci 32, 20133 Milan, Italy; lucia.donno@polimi.it (L.D.); cecilia.monoli@taltech.ee (C.M.); carlo.frigo@polimi.it (C.A.F.); 2 Department of Computer System, Tallinn University of Technology, Ehitajate tee 5, 12616 Tallinn, Estonia

**Keywords:** backward walking, forward walking, gait analysis, kinematics, kinetics, optoelectronic system, force plates

## Abstract

Although extensive literature exists on forward and backward walking, a comprehensive assessment of gait parameters on a wide and homogenous population is missing. Thus, the purpose of this study is to analyse the differences between the two gait typologies on a relatively large sample. Twenty-four healthy young adults participated in this study. By means of a marker-based optoelectronic system and force platforms, differences between forward and backward walking were outlined in terms of kinematics and kinetics. Statistically, significant differences were observed in most of the spatial–temporal parameters, evidencing some adaptation mechanisms in backward walking. Differently from the ankle joint, the hip and knee range of motion was significantly reduced when switching from forward to backward walking. In terms of kinetics, hip and ankle moment patterns for forward and backward walking were approximately mirrored images of each other. Moreover, joint powers appeared drastically reduced during reversed gait. Specifically, valuable differences in terms of produced and absorbed joint powers between forward and backward walking were pointed out. The outcomes of this study could represent a useful reference data for future investigation evaluating the efficacy of backward walking as a rehabilitation tool for pathological subjects.

## 1. Introduction

Walking is an essential form of human locomotion and it is undoubtedly the most investigated motor task because of its tight connection with health status and quality of life. A proper gait pattern allows for efficient movement and minimization of fatigue, while gait abnormalities are often associated with problems of the locomotor system or neurological dysfunctions. These conditions, in turn, can lead to musculoskeletal complications, such as back and joint pain or foot diseases [1,2,3]. Locomotion plays a crucial role in maintaining physical fitness and health in general, to the point that several clinical functional scales have been proposed [4]. Some examples are the EVGS [5] and GMFCS [6] for children with Cerebral Palsy, the POMA scale for assessing older adults and predicting their propensity to fall [7], the FES-DMD-GD for individuals with Duchenne Muscular Dystrophy [8], the UPDRS [9] for patients with Parkinson disease, and the GABS scale [10] for a comprehensive gait and balance assessment. The scientific investigation and quantitative analysis of locomotion (gait analysis) are important both for clinical diagnosis and monitoring the effects of treatments. The identification of gait pattern abnormalities can help detect functional limitations and their possible causes, so that specific treatment interventions and rehabilitation protocols can be defined. This applies to both the clinical and orthopaedic environment, as well as the sports science fields [11,12].

Given the fundamental importance of walking in everyday activities and its tight connection with well-being, gait training is frequently part of the rehabilitation protocols. Forward Walking (FW) can easily be performed by individuals of all ages and fitness levels, and it is commonly indicated for patients recovering from injuries and surgeries at the lower limbs, suffering from chronic conditions, disabilities and neurodegenerative disorders [13]. More specifically, walking helps preserving muscular strength and balance control, coordination and proprioception, which can reduce the risk of falls and injuries. The activity of walking helps in weight management by burning calories and increasing metabolism [14]. At cardiovascular level it is beneficial in strengthening the heart and lowering blood pressure [15]. Finally, gait training has been shown to have mental health benefits, such as reducing stress and anxiety [16,17]. These are all fundamental factors to help patients regain independence after an injury or to overall improve their well-being and quality of life.

On the other hand, Backward Walking (BW) is not frequently included in rehabilitation protocols, although it enhances balance and muscle strength [18]. Moreover, BW could have various beneficial effects on body systems in terms of reducing pain symptoms, improving joint range of motion and overall stability [19], and generating important factors to enhance well-being and physical recovery. BW training is particularly suitable for individuals who are unable to engage in high-impact exercises [20]. Walsh et al. [21] investigated the complexity and the symmetry of backward gait pointing out the greater variability of BW for temporal parameters, joint angles and muscular activation. Long-term BW training has been also shown to have beneficial effects on spatial–temporal gait parameters and balance ability, as reviewed by Wang et al. [20,22]. Considering the outcomes of BW training on abnormal gait, Choi et al. [17] investigated the effects of reversed walking training on balance and locomotion capacities in hemiplegic Cerebral Palsy children, observing great improvements in stability and gait parameters. These beneficial effects were even larger than those observed after ordinary forward gait training. Similar valuable outcomes were observed on residual walking capacities by Michaelsen et al. [18] when comparing the effects of forward and backward treadmill training on people after a stroke. Interestingly, the importance of BW has been also pointed out by DeMark et al. [23] that recently proposed a variant of the 3 m walk test. This well-established tool for physical activity and deambulatory skills assessment was performed during walking backward and proposed as an assessment facility for motor skills and post-stroke rehabilitation.

To date, the differences between FW and BW have been broadly discussed. Winter et al. [24] compared the two motor tasks and considered BW as a simple temporal reversal of FW determining that the same group of neurons might be controlling them since the walking patterns are kinematically similar but performed in reverse of time. Deursen et al. [25] also pointed out that the muscular activation patterns during BW suffer from a phase shift of approximately 25%, accountable by the adaptation of the control mechanism. Zhang et al. [19] dedicated attention to the knee external abduction moment during BW, pointing out that this motor task reduced the joint loading and confirmed its potentiality in the management of knee osteoarthritis. Milane et al. [13] showed that BW challenges the body differently than FW, stimulating balance and coordination in a more demanding way, and improving cognitive function, proprioception and spatial awareness. Dissimilarities between FW and BW kinematics have been also identified in people with intellectual disabilities by Gimunova et al. [26]. The cited authors observed reduced velocity and step length in BW regardless of the intellectual impairment, although a greater reduction was appreciable in subjects with Down syndrome.

Despite the extensive literature existing on forward and backward walking, the previous studies are mostly focused on specific gait parameters or involved a limited number of participants. Our work is aimed at overcoming these limits by collecting data on a relatively wide population of healthy volunteers and by deeply investigating the two motor tasks by means of motion capture optoelectronic system and force plates. The final aim of this investigation is two-fold: (i) to analyse the differences between forward and backward walking in terms of kinematics and kinetics on a homogeneous sample of male and female young adults; and (ii) to define common behaviour and patterns in healthy young adults when performing the two tasks, and thus providing reference data for comparison with pathological patterns.

## 2. Materials and Methods

### 2.1. Participants

Twenty-four healthy subjects, 12 females and 12 males, participated in this study. The sample had a mean age of 26.13 ± 2.03 years, a mean height of 1.73 ± 0.08 m, a mean body mass of 64.88 ± 11.37 kg, and a mean BMI of 21.52 ± 2.61 kg/m^2^ (Table 1).

The sample consisted of participants who were healthy with an age between 23 and 30 years and able to walk without any assistive device. Subjects with a history of injuries to lower limbs, lower limb deformities, musculoskeletal or neurological disorders were excluded from the study.

All participants were required to sign a written informed consent form, in which the experimental tests were described in detail. The study was carried out in compliance with the World Medical Association Declaration of Helsinki and approved by the Ethics Committee of Politecnico di Milano.

### 2.2. Instrumentation

For each participant, gait data and anthropometric parameters were collected in the MovLab “Luigi Divieti” laboratory at Politecnico di Milano, Milan, Italy. Kinematic data were recorded by means of 8-infrared digital cameras connected to a motion capture system (SMART-DX 400, BTS Bioengineering, Garbagnate Milanese, Milan, Italy) with a sampling rate of 100 Hz and a measurement accuracy lower than 0.3 mm. Regarding the technical specifications of the optoelectronic system, each camera featured highly sensitive sensors with a resolution of 1366 × 768 (1 M pixel), and functional LED illuminators with a wavelength of 850 nm. Two OPTIMA HPS force platforms (AMTI, Watertown, MA, USA) placed approximately in the centre of the walkway were used for the acquisition of the ground reaction forces (GRF) of each foot during the stance phase of walking. Each force plate was 400 × 600 × 82.5 mm^3^ with a sampling frequency of 500 Hz and equipped with six channels supplied with a voltage of 5 V. Such device ensured an accuracy of less than 0.2 mm in estimating the position of the Centre Of Pressure (COP) and a force measurement accuracy of ±0.1% of applied load. Two TV cameras synchronized with the optoelectronic system were employed for videorecording frontal and sagittal views.

### 2.3. Procedures

On each subject, the following anthropometric parameters were measured, in accordance with the Davis [27] protocol: height, body mass, pelvis width (measured as anterior superior iliac spines-ASIS-distance), pelvis height (great trochanter to ipsilateral ASIS distance), knee diameter (distance between medial and lateral femoral epicondyles), ankle diameter (medial to lateral malleoli distance) and lower limb length (distance between ASIS and ipsilateral medial malleolus). Then, 22 spherical infrared reflective markers were attached on the skin overlying specific anatomical landmarks according with the Davis protocol [27]. It is worth mentioning that one operator was responsible for the markers’ positioning and data acquisition to limit the inter-individual errors and to ensure consistency. Subjects, in shorts and barefoot, were instructed to walk forward along a 10 m straight walkway at their natural cadence. The task was repeated until 5 valid FW trials were obtained for each subject. A trial was considered valid if the following conditions were met: a good quality of the markers’ trajectory and one foot in full contact with each force platform. Then, the subject was asked to walk backward keeping their head and upper body in a natural straight position. Five valid BW trials were acquired for each volunteer. In view of the difficulty of walking backwards and the consequent possible fatigue, a one-minute break between trials was granted.

### 2.4. Data Analysis

Collected data were processed by the dedicated software SMART Clinic (BTS Bioengineering, Garbagnate Milanese, Milan, Italy) for assessing kinematic and kinetic parameters. Specifically, kinematics was analysed in terms of spatial–temporal parameters and joint angles on a sagittal plane. Hip, knee and ankle joints kinetics were computed in terms of moments and powers. Moreover, three-dimensional ground reaction forces were analysed.

**Figure 1:**
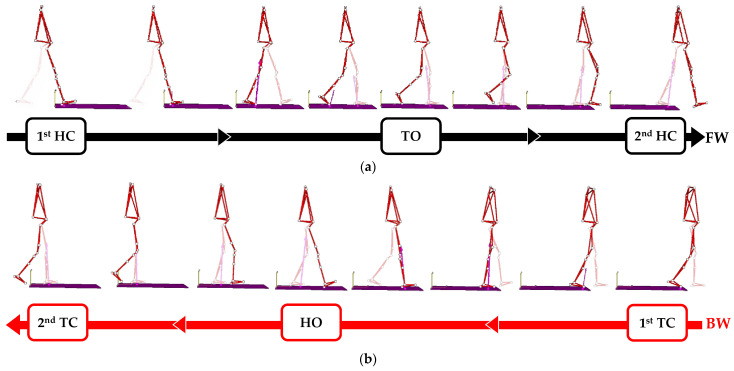
Definition of the gait cycle. Black and red arrows represent the direction of progression during walking. The purple vector displays the GRF. Gait events are referred to the right lower limb (the contralateral limb is colourless). (**a**) Forward walking: from the first to the second heel contacts, toe off marks the onset of the swing phase. (**b**) Backward walking: from the first to the second toe contacts, heel off marks the onset of the swing phase.

Kinematic and kinetic patterns were time normalised on the gait cycle, specifically defined for FW and BW. According to tradition, the gait cycle of FW was set as the period between the initial contact of one foot on the ground to the subsequent initial contact of the same foot (Figure 1). Hence, the stance phase of the FW gait cycle was defined as the interval between the initial contact (hereinafter referred to as “heel contact” since all subjects approached the ground by the heel) and the toe off. Accordingly, for each lower limb, the swing phase was marked between the toe off event and the second heel contact.

Consistently, the BW gait cycle was defined as the interval between two subsequent initial contacts, which all volunteers freely performed by approaching the toe to the ground (hereinafter referred to as “toe contact”). During the BW gait cycle, at the end of the stance phase, the participants initiated the swing phase by detaching the heel from the ground (“heel off”). In this sense, in the comparisons of all variables, the heel contact (HC) for forward walking matched the toe contact (TC) for backward walking, while the toe off (TO) of FW corresponded to the heel off (HO) of BW, as depicted in Figure 1.

For each subject, the ground reaction forces, joint moments and powers were normalised with respect to body mass. Males and females were ensemble and included in the same sample, since no statistical difference was found between their BMI index (*p* = 0.065). Moreover, patterns of joint angles, moments and powers were ensemble and averaged across the 48 lower limbs, since all the involved subjects were healthy and unaffected by specific asymmetries and the study was not intended to investigate laterality effects. Thus, considering 5 valid trials for each lower limb, 240 acquisitions were averaged for each variable.

Spatial and temporal parameters were quantified for both FW and BW trials. Average speed and step width were averaged across the 5 trials of the 24 participants, thus their mean value resulted from the average of 120 recorded parameters. Instead, the mean value of stance and swing phase duration, double support phase duration, stride and step length, which are referred to each limb, resulted from the average of 240 parameters. Moreover, Range Of Motion (ROM) on the sagittal plane of hip, knee and ankle during FW and BW were quantified for each trial and ensemble averaged across 240 values for each task typology.

### 2.5. Statistical Analysis

The statistical analysis was performed in Minitab^®^ (version 21.3.1, State College, PA, USA). Specifically, the data normality was tested applying the Kolmogorov–Smirnov test. It showed that spatial and temporal parameters were not normally distributed (*p* < 0.05). Hence, to compare the differences in terms of gait spatial–temporal parameters among FW and BW, a non-parametric analysis was performed (Mann–Whitney test). A statistically significant difference was accepted when *p* ≤ 0.05. Regarding ROM parameters, the Kolmogorov–Smirnov test positively verified their normal distribution. Thus, their comparison between FW and BW was performed by the two-tailed t-Student’s test (α ≤ 0.05).

## 3. Results

For the sake of clarity, the outcomes concerning spatial–temporal parameters, joint kinematics and kinetics patterns are outlined separately.

### 3.1. Spatial–Temporal Parameters

In Table 2 the median, 1st and 3rd quartiles of the main spatial–temporal parameters are reported. The first evident difference between FW and BW was the average speed of progression (*p* < 0.001), whose median value was 1.1 m/s and 0.8 m/s, respectively. 

Interestingly, for 50% of the sample, the average speed was between 1 m/s and 1.2 m/s, while 1.1 m/s was the maximum average speed reached in only few trials during BW. Specifically, in BW just 25% of the sample reached an average speed higher than 0.9 m/s. In Table 2, the average speed is also reported showing normalised with respect to the subject’s height (Normalised Average speed).

The median of stance phase duration was 61.75% and 60.77% of the FW and BW gait cycle (*p* < 0.001), respectively. Consistently, the duration of the swing phase occupied the 38.25% of the FW cycle and the 39.22% of BW cycle (*p* < 0.001). Referring to the gait cycle duration, the double support phase was quite longer during FW with respect to BW (*p* < 0.001).

The stride length, defined as the distance between two consecutive contacts with the same foot, was measured both in meters and as percentage of the subject’s height (Normalised Stride length). 

As depicted in Table 2, during BW, the stride length (65.35% of height) was shorter than in FW, in which the median value was 72.43% of the height (*p* < 0.001). The step length, defined as the distance between two consecutive contacts of contralateral feet, was consistently smaller during BW with respect to FW (*p* < 0.001). Finally, the step width, defined as the transversal distance between the two feet during double support, showed a significant statistical difference (*p* < 0.001): the median value was larger in BW (0.14 m) compared to FW (0.09 m).

### 3.2. Kinematics

**Figure 2:**
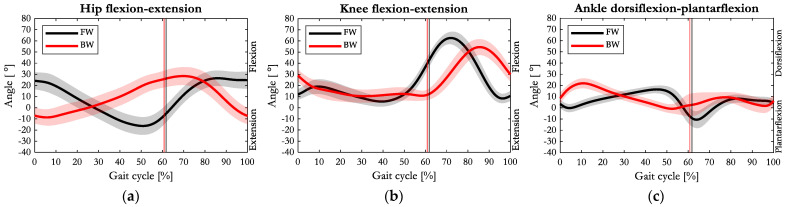
Average (solid line) and one standard deviation of angle patterns of (**a**) hip, (**b**) knee, and (**c**) ankle joints on the sagittal plane during FW (black) and BW (red). Vertical lines represent the toe off event of FW (black) and the heel off event of BW (red).

The joints’ angle average patterns of FW and BW are depicted in Figure 2. Specifically, 0% and 100% of the gait cycle marked the two consecutive HCs for FW and the two sequential TCs for BW, respectively. The TO and the HO events (black and red vertical lines in figures) marked the beginning of the swing phase for FW and BW, respectively.

Conversely to FW, in BW, the hip joint appeared slightly extended (8.6°) at the beginning and at the end of the gait cycle. Furthermore, during BW the hip reversed its pattern from the initial contact up to the terminal stance phase (approximately 50% of gait cycle). As highlighted in Figure 2a, while the hip reached the largest extension (16.3°) during FW, in BW, it kept flexing until 70% of the gait cycle, where a mean maximum flexion angle of 28.4° was found.

As depicted in Figure 2b, minor changes were recorded on the knee joint angle pattern between FW and BW. A difference appeared at the initial contact, where in BW, the knee flexed by 30° and suddenly extended, while in FW it flexed by only 10° and then it flexed (load acceptance). During the swing-phase, a temporal shift was appreciable. In particular, during BW, the knee reached its maximum flexion (54.5°) with a delay of approximately 14% of the gait cycle with respect to FW, where a maximum flexion of 62.7° was recorded. As reported in Figure 2c, when switching from FW to BW, the ankle joint pattern appeared reversed up to the 50% of the gait cycle, in that the normal sequence of plantarflexion–dorsiflexion was substituted by a dorsiflexion–plantarflexion cycle. With respect to FW, in BW, the ankle plantarflexion in the pre-swing phase (from 50% to 60% of the gait cycle) was considerably reduced.

**Figure 3:**
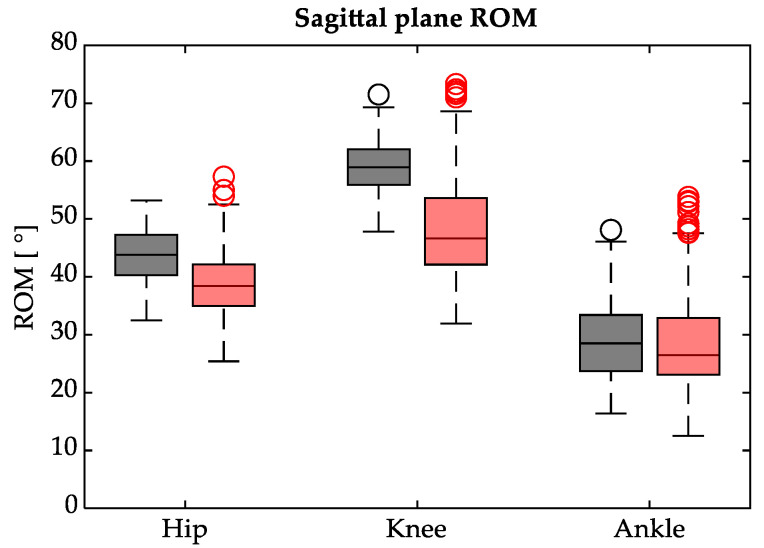
ROM on the sagittal plane of hip, knee and ankle joints during FW (black) and BW (red).

On the sagittal plane, the hip, knee and ankle ROMs were calculated as the difference between the maximum and minimum value in the flexion–extension pattern (Figure 3). 

**Figure 4:**
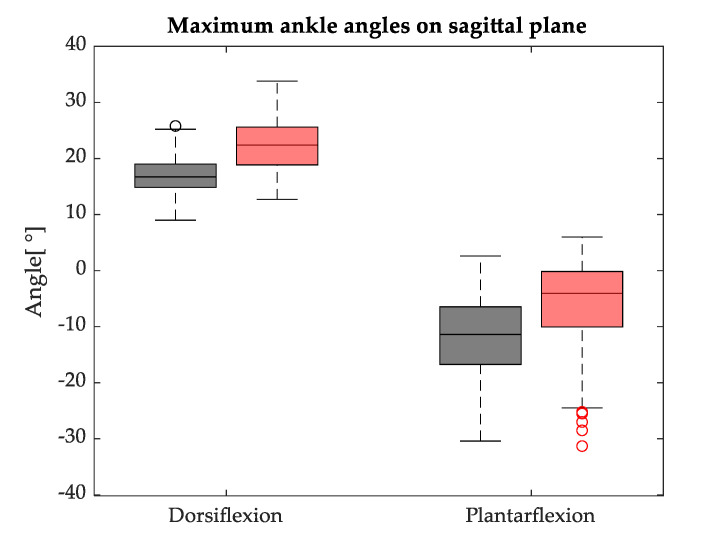
Maximum plantarflexion and dorsiflexion angles reached during FW (black) and BW (red).

On average, the hip joint reached a wider ROM during FW (44.0 ± 4.7°) with respect to BW (39.0 ± 5.66°). This difference proved to be statistically significant, since *p* < 0.001. Similarly, the mean knee ROM showed a significant statistical difference (*p* < 0.001): it was larger during FW (approximately 58.8 ± 4.5°) than in BW (47.9 ± 8.5°). Conversely, no statistical difference was found on the mean ROM of the ankle joint (*p* > 0.05): it was 28.9 ± 6.8° during FW and 28.5 ± 8.4° in BW. However, when the maximum plantarflexion and maximum dorsiflexion were analysed separately (Figure 4), statistical differences (*p* < 0.001) between FW and BW appeared for both parameters: on average, the maximum dorsiflexion was greater during BW (22.3 ± 4.4°) compared to FW (16.9 ± 3.4°), while the maximum plantarflexion angle was larger in FW (11.9 ± 7.4°) than in BW (6.2 ± 7.4°).

### 3.3. Kinetics

**Figure 5:**
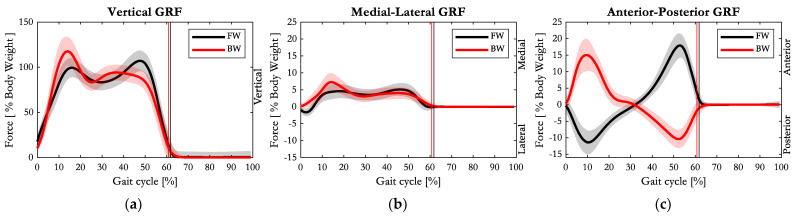
Average (solid line) and one standard deviation of (**a**) vertical, (**b**) medial–lateral, and (**c**) anterior–posterior components of GRF during FW (black) and BW (red). Vertical lines represent the toe off event of FW (black) and the heel off event of BW (red).

In Figure 5, the vertical, medial–lateral and anterior–posterior components of the GRF, expressed as percentage of body weight, are depicted. 

The vertical component of GRF maintained the characteristic double bump, but its trend appeared switched: during FW, the maximum peak of force was the second one (108 ± 6.3% of body weight, at approximately the 50% of the gait cycle), while during BW, the highest peak was the first one (118 ± 15.9% of body weight at 15% of the gait cycle). In correspondence with the first peak, the medial component of GRF was also larger in BW (7.3 ± 2.7% of body weight) than in FW (4.6 ± 2.1% of body weight). 

The pattern of the anterior–posterior component of GRF during BW was approximately the mirror image of the FW one: with respect to the laboratory absolute reference frame, during FW, the GRF inclination gradually changed from posterior to anterior as the COP moved from the heel that performed the first contact to the forefoot, making the toe-off. Conversely, during BW, the GRF was directed forward at the initial contact performed by the forefoot and progressively directed posteriorly as the COP moved towards the heel, performing the heel off. In both FW and BW, the first part of the stance phase corresponds to load absorption (or braking phase), while the second part is associated with push off (acceleration of the body mass in the direction of walking). The peak of the braking phase in BW was higher (in absolute value) than the peak in FW (15.0 ± 4.9% versus 11.4 ± 3.6% of body weight, *p* < 0.001), while the peak of push off was smaller in BW than in FW (10.4 ± 2.8% versus 17.9 ± 3.6% of body weight, *p* < 0.001).

**Figure 6:**
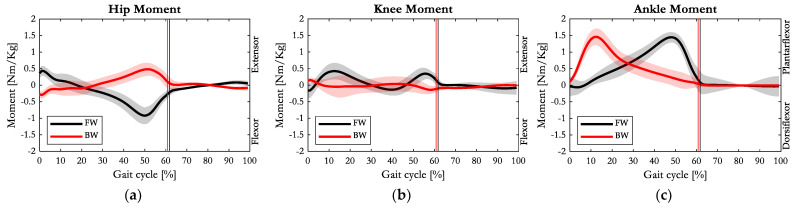
Average (solid line) and one standard deviation of internal moment patterns of (**a**) hip, (**b**) knee, and (**c**) ankle joints on the sagittal plane during FW (black) and BW (red). Vertical lines represent the toe off event of FW (black) and the heel off event of BW (red).

The averaged joint moment patterns normalised with respect to body mass, are depicted in Figure 6. 

The hip moment patterns for FW and BW were approximately mirrored images of each other: during BW, the external moment changed from extensor (in early stance phase) to flexor up to the pre-swing phase. In the phase preceding the swing phase, approximately at 50% of the gait cycle, the hip joint reached the maximum external extensor moment during BW (0.48 Nm/kg on average), and vice versa, the maximum external flexor moment (mean value of 0.92 Nm/kg) during FW.

With respect to FW, the extensor moment requested at the knee joint was drastically reduced during BW: its maximum value occurred at the initial contact (0.15 Nm/kg) when the knee was slightly flexed (Figure 2b), and thus its joint centre was in a position just ahead of the GRF vector. A similar condition generated an external flexor moment (0.14 Nm/kg) at approximately 40% of the gait cycle. Then, throughout the gait cycle, the GRF vector maintained an anterior position with respect to the knee joint centre (external extensor moment). 

Consistently with the BW characteristic (the COP progression from the forefoot to the heel), the ankle experienced the largest external dorsiflexion moment at the early stance, approximately at 10% of the gait cycle. This resulted from the combination of high force and a large lever arm, and at this time instant, the GRF reached both its largest anterior and vertical component (Figure 5a,c), and the ankle was at its maximum dorsiflexed configuration, as evident from Figure 2c. The same ‘external dorsiflexion’ moment took place in the push-off phase during FW. Specifically, in the two gait typologies, the maximum external dorsiflexor moment at the ankle joint were equivalent (about 1.45 Nm/kg, as visible in Figure 6c).

**Figure 7:**
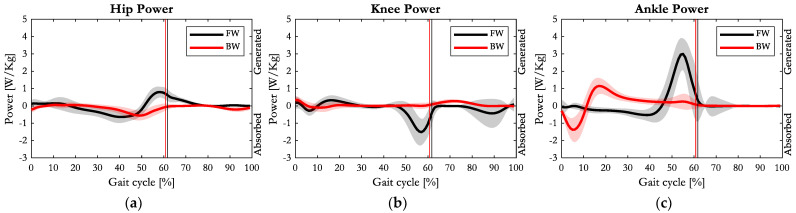
Average (solid line) and one standard deviation of power patterns of (**a**) hip, (**b**) knee, and (**c**) ankle joints on the sagittal plane during FW (black) and BW (red). Vertical lines represent the toe off event of FW (black) and the heel off event of BW (red).

Figure 7 provides the power patterns of hip, knee and ankle joints recorded during FW and BW, normalised with respect to body mass.

At first glance, it was evident that the power was considerably reduced on all the considered joints when switching from FW to BW. Specifically, during FW, the hip joint performed an absorption–generation power cycle during the late stance pre-swing phase of the gait cycle, whereas in BW the power generation phase was absent, and just a peak of power absorption was evident in correspondence with the maximum joint moment. The maximum absorbed power during BW (0.56 W/kg) was slightly smaller than the one recorded during FW (0.64 W/kg). 

Regarding the knee joint, a short phase of power generation appeared at the initial contact (0.34 W/kg) during BW. This value was slightly higher than the initial power generation measured at the heel contact (HC) during FW (0.19 W/kg). During the early stance phase, in FW, a cycle of power absorption–generation was evident, while in BW just a small absorption appeared (0.1 W/kg). In the pre-swing and late-swing phase of the FW gait cycle, two peaks of power absorption were evident. Conversely, in these phases of the BW gait cycle, the knee power was almost zero. Only in the early swing phase, a small peak of power generation appeared (0.28 W/kg), while in FW it was nil.

At the ankle joint, consistently with the small inertial characteristics, both FW and BW powers and moments were almost null during the swing phase, whereas interesting patterns were recorded during the stance phase. In BW, after a short-lasting absorption (1.36 W/kg) at the initial contact (TC), a peak of power (1.14 W/kg) was generated during the early stance phase. Then, a long-lasting power generation was evident up to the HO event. Conversely, in FW, after an enduring power absorption, a higher peak of power (3 W/kg) was generated in preparation for the swing phase.

## 4. Discussion

The present study aimed to establish reference data of forward and backward gait, considering spatial–temporal parameters, kinematics and kinetics data. The outcomes were overall in agreement with previous studies. 

### 4.1. Spatial–Temporal Parameters

Specifically, in accordance with Zhang et al. [19], a reduction of walking speed during BW of about 27% with respect to FW was observed. Table 2 also pointed out that the median stance and swing phases, expressed as a percentage of the gait cycle, were similar for the two tasks [25]. Additionally, it is worth noting that the BW parameters have an overall variability larger than in FW, and this appears from the width of the interquartile range. This observation is also in agreement with Winter et al. [24], who related the higher unpredictability of BW to the absence of visual feedback and to the unpractised and non-use of this motor task. This was further supported by the measured larger step width, shorter stride and step lengths, and evidence that the participants needed more stability and balance while performing this unusual task. 

### 4.2. Kinematics

Observing the kinematic data collected, in accordance with [25], it was noticeable that the hip and knee range of motion decreased significantly during BW, while the ankle ROM remained comparable to FW. Furthermore, by observing the dispersion of the boxplots around the median in Figure 3, an analysis similar to the one made for the spatial–temporal parameters could be performed, confirming the lower repeatability of the BW task with respect to FW. The joint angles of the hip, knee and ankle, normalised over the gait cycle for FW and BW in Figure 2, were consistent with the previous analysis [25] that pointed out how during BW, the knee appears to be less involved in the stance phase of the gait cycle (before the heel-off) with a consequent delay in the flexion pattern. Interestingly, hip and ankle joint angles patterns during BW appeared upside down with respect to forward gait. 

### 4.3. Kinetics

The measured joint moments (Figure 6) appeared almost perfectly reversed for the hip and ankle in the first half of the gait cycle, while for the knee, a much smaller magnitude and an external flexor moment at the toe contact event in BW was observed. Then, except for the initial contact and 40% point of the gait cycle, the GRF vector maintained an anterior position with respect to the knee joint centre (external extensor moment). Moreover, with respect to the knee joint centre the lever arm of the GRF was kept quite short: despite the progressive increment of the GRF posterior component, the knee joint centre was maintained posteriorly to the force vector thanks to the persistent extension, causing almost null flexor knee moment. These observations are consistent with Winter et al. [24] and in accordance with the review conducted by Wang et al. assessing the beneficial effect of BW in reducing knee joint loads [22]. 

Further important observations can be drawn from the vertical GRF data in Figure 5a, consistent with Zhang et al. [19]. While previous studies on FW highlighted that a reduced walking speed corresponds to a smaller first peak of the GRF [28], our investigation points out that this behaviour is reversed during BW, assessing a higher peak of vertical GRF in the initial early stance phase despite the lower speed of progression. The medial–lateral GRF component, for both FW and BW, was consistently pointing medially toward the centre of gravity, ensuring gait stability. A slightly bigger magnitude of medial GRF during the early stance phase of BW was substantial, and probably related to the increased step width, resulting in a bigger adduction moment at the hip than in FW. The GRF inclination on the sagittal plane followed the walking direction: going from posterior to anterior in FW, and vice versa in BW.

Regarding the joint power outcomes, it was evident at first glance that they were significantly reduced when switching from FW to BW. This is consistent with the lower average progression speed recorded during BW compared to FW. The time course of joint power represents the sequence of energy production and absorption: positive values (power produced) are mainly associated with the concentric activity of muscles, while negative values (power absorbed) may be due to energy absorbed by either muscles or passive structures. The energy, being the time integral of power, corresponds to the area subtended by the power curve, representing the work produced (if the area is in the positive hemi-plane) or absorbed (area in the negative hemi-plane). With reference to this, the power trend at the ankle joint showed an interesting behaviour during BW, in which an absorption–generation cycle of energy was evident during the early stance phase (Figure 7c). This mechanism is advantageous in terms of total energy expenditure, since it implies that the energy produced does not necessarily originate from metabolic energy, but could be partly derived from the restitution of energy stored in the form of elastic energy in the musculotendinous tissues operating at the ankle. Specifically, in BW, the ankle plantarflexor muscles are activated just after the initial contact, in order to contrast the external dorsiflexor moment, and are stretched to allow for dorsiflexion of the ankle (load acceptance). Thus, in this interval the triceps surae absorbs energy, and we see power absorption in the first 10% of the gait cycle. Then, plantarflexors contraction becomes concentric (ankle plantarflexion, positive power) until the pre-swing phase. In this interval, it is possible that part of the previously absorbed energy, stored in the passive structures, can be released for power generation. In FW, after the initial contact, the amount of energy absorption is quite small due to a small plantarflexor moment and a small angular variation. Conversely, in BW, the absorbed energy after the initial contact is quite large, corresponding to a great plantarflexor moment that responds to the external load generated by the GRF while applied on the forefoot and to the wide variation of the ankle angle. Hence, while during FW the triceps surae is one of the major responsible muscles for power generation at the ankle during the push-off phase, and in BW it becomes the principal absorber of power at the initial contact to ensure a controlled landing on the ground during the load acceptance phase. This finding was in agreement with Winter et al. [24].

Regarding the knee joint, considering both the moments and powers entities, BW appeared to require a lower muscular effort than FW. The typical cycle of power absorption-production during the load acceptance phase in FW was almost non-existent in BW. Contrarily to FW, at the initial toe contact in BW, the knee extended against the external flexor moment resulting in positive power generated by the quadriceps muscles. Then, during the load acceptance phase, the extensor muscles undergo an elongation-shortening cycle (absorption–generation of power) in FW, whereas in BW just a small absorption of power was evident: the knee joint extended under the small external extensor moment; hence, the hamstrings muscle group (flexors of the knee) could absorb energy in this phase and return it back during the early swing phase (power generation) to produce the knee flexion. Therefore, during the load acceptance phase, in FW the knee extensors are the main responsible for energy absorption, whereas in the backward gait this role is played by the antagonist muscles, as also supported by the activation patterns recorded by Winter et al. [24]. 

The power time course at the hip joint during FW showed the typical cycle of power absorption–generation occurring after 20% of the gait cycle. From 20% to 60% of the gait cycle, the hip external moment is extensor, while the hip angular movement switches from extension to flexion at 50% of the gait cycle. As a consequence, the power is absorbed in the first phase (before 50% of the stride cycle with a negative peak at about 40%), and produced in the second phase (from 50% to 60% of the stride cycle). Compared to FW, the BW hip moment appeared as a mirror image resulting in long-lasting power absorption (from 30% to 60% of the gait cycle). However, conversely to FW, there was no power generation following the absorption phase. In fact, in BW, the hip external moment was flexor for the whole time, but differed with FW, as the hip did not switch its movement at 50% of the stride cycle, and instead kept flexing until the end of the stance phase and early swing. Because of this, the extensor muscles, which counteract the flexor moment, contract eccentrically and the power is absorbed. 

## 5. Conclusions

Despite the variety of studies on forward and backward walking, a global overview of the kinematic and kinetic differences between the two gait typologies on a wide and homogeneous sample was missing. Knowing these differences could be useful for clinicians to consciously include backward walking as a training activity during rehabilitation, by evaluating whether the kinematic and kinetic characteristics of this motor task could be appropriate and beneficial for a specific subject. In general, backward walking data showed higher variability with respect to forward walking, probably due to the absence of visual feedback and to the unusual motor task. Interesting adaptation mechanisms in terms of spatial–temporal parameters in backward walking were outlined: aiming for stability, the individuals adopted a larger step width and shorter stride and step lengths. Furthermore, from the initial contact up to the terminal stance phase, hip and ankle joint angles patterns during backward walking appeared upside down with respect to forward gait. Moreover, differences in terms of produced and absorbed joint powers, as well as muscular involvement, were pointed out. Therefore, a useful reference frame for future studies on forward and backward gait parameters involving a homogeneous sample of young adults was defined. However, in order to draw a more general conclusion and to provide a comprehensive reference for the comparison with a wider population, future studies should involve different age-groups (i.e., paediatric and elderly ranges).

## Figures and Tables

**Table 1:** General characteristics of participants.

Participants	Age [years]	Body Height [m]	Body Mass [kg]	BMI [kg/m^2^]
12 Females	26.03 ± 1.96	1.66 ± 0.04	56.51 ± 9.06	20.49 ± 3.27
12 Males	26.23 ± 2.19	1.80 ± 0.05	73.25 ± 5.96	22.54 ± 1.14
Sample	26.13 ± 2.03	1.73 ± 0.08	64.88 ± 11.37	21.52 ± 2.61

**Table 2:** Spatial–temporal parameters in FW and BW. * *p* < 0.001.

		FW			BW		
		Median	1st Q	3rd Q	Median	1st Q	3rd Q
**Average speed**	[m/s]	1.1	1	1.2	0.8 *	0.8	0.9
**Normalised Average speed**	[% height/s]	62.45	58.89	67.81	49.14 *	44.4	52.7
**Stance phase**	[% gait cycle]	61.75	60.69	62.92	60.77 *	59.52	62.76
**Swing phase**	[% gait cycle]	38.25	37.08	39.3	39.22 *	37.23	40.48
**Double support phase**	[% gait cycle]	11.76	10.62	12.82	10.86 *	9.58	12.68
**Stride length**	[m]	1.25	1.19	1.31	1.12 *	1.06	1.19
**Normalised Stride length**	[% height]	72.43	68.94	75.43	65.35 *	61.16	68.96
**Step length**	[m]	0.62	0.59	0.66	0.56 *	0.52	0.6
**Step width**	[m]	0.09	0.07	0.11	0.14 *	0.11	0.17

## Data Availability

Data available on request due to restrictions, e.g., privacy or ethical.
